# Web-based gene expression analysis—paving the way to decode healthy and diseased ocular tissue

**DOI:** 10.1007/s00347-022-01721-4

**Published:** 2022-09-13

**Authors:** Julian Wolf, Thabo Lapp, Thomas Reinhard, Hansjürgen Agostini, Günther Schlunck, Clemens Lange

**Affiliations:** 1 Eye Center, Medical Center, Faculty of Medicine, University of Freiburg, Freiburg, Germany; 2 Ophtha-Lab, Department of Ophthalmology, St. Franziskus Hospital Muenster, Münster, Germany

**Keywords:** Transcriptome, RNA-Seq, Database, Eye, Biomarker, Transkriptom, RNA-Sequenzierung, Datenbank, Auge, Biomarker

## Abstract

**Background:**

Gene expression analysis using RNA sequencing has helped to improve the understanding of many diseases. Databases, such as the *Gene Expression Omnibus database* of the *National Center for Biotechnology Information* provide RNA sequencing raw data from various diseased tissue types but their analysis requires advanced bioinformatics skills. Therefore, specific ocular databases provide the transcriptional profiles of different ocular tissues and in addition enable intuitive web-based data analysis.

**Objective:**

The aim of this narrative review is to provide an overview of ocular transcriptome databases and to compare them with the *Human Eye Transcriptome Atlas* newly established in Freiburg.

**Methods:**

PubMed literature search.

**Results:**

A total of nine ocular transcriptome databases focusing on different aspects were identified. The *iSyTE* and *Express* platforms specialize in gene expression during lens and retinal development in mice, whereas *retina.tigem.it*, *Eye in a Disk*, and *Spectacle* focus on selected ocular tissues such as the retina. *Spectacle*, *UCSC Cell Browser* and *Single Cell Portal* allow intuitive exploration of single cell RNA sequencing data derived from retinal, choroid, cornea, iris, trabecular meshwork and sclera specimens. The microarray profiles of a variety of healthy ocular tissues are included in the *Ocular Tissue Database*. The *Human Eye Transcriptome Atlas* provides the largest collection of different ocular tissue types, contains the highest number of ocular diseases and is characterized by a high level of quality achieved by methodological consistency.

**Conclusion:**

Ocular transcriptome databases provide comprehensive and intuitive insights into the transcriptional profiles of a variety of healthy and diseased ocular tissues. Thus, they improve our understanding of the underlying molecular mediators, support hypothesis generation and help in the search for new diagnostic and therapeutic targets for various ocular diseases.

## Abstract (de)

**Hintergrund:**

Die Entschlüsselung des Transkriptoms hat in den letzten Jahren unser Verständnis zahlreicher Erkrankungen verbessert. Öffentlich zugängliche Datenbanken, wie z. B. die *Gene Expression Omnibus*-Datenbank des *National Center for Biotechnology Information*, sammeln Transkriptomrohdaten aus einer Vielfalt von Proben, ohne jedoch dem bioinformatischen Laien einen intuitiven Zugang zu den Daten zu gewähren. Daher wurden in den vergangenen Jahren spezielle Transkriptomdatenbanken programmiert, die eine benutzerfreundliche Web-basierte Datenanalyse ermöglichen und damit niederschwellig molekulare Einblicke in okuläre Gewebe ermöglichen.

**Fragestellung:**

Ziel dieser Arbeit ist es, einen Überblick über die aktuell verfügbaren okulären Transkriptomdatenbanken zu geben und diese mit dem in Freiburg neu etablierten *Human Eye Transcriptome Atlas* zu vergleichen.

**Methoden:**

Literatursuche in PubMed.

**Ergebnisse:**

Neun okuläre Transkriptomdatenbanken mit unterschiedlichem Anwendungsschwerpunkt wurden identifiziert. Die Plattformen *iSyTE* und *Express *spezialisieren sich auf die Genexpression während der Linsen- und Netzhautentwicklung der Maus, wohingegen *retina.tigem.it, Eye in a Disk* und *Spectacle* ihren Fokus auf einzelne okuläre Gewebe wie die Netzhaut legen. *Spectacle, UCSC Cell Browser *und *Single Cell Portal *erlauben die intuitive Exploration von Einzelzell-RNA-Sequenzierungsdaten von Netzhaut‑, Aderhaut‑, Kornea‑, Iris‑, Trabekelmaschenwerk- und Skleragewebe. Die Microarray-Profile verschiedener gesunder okulärer Gewebe werden in der *Ocular Tissue Database *bereitgestellt. Der *Human Eye Transcriptome Atlas* erfasst derzeit die größte Vielfalt an Augengeweben und Erkrankungen des Auges. Er zeichnet sich durch einen hohen Qualitätsstandard aus, der durch methodische Homogenität erreicht wird.

**Schlussfolgerungen:**

Okuläre Transkriptomdatenbanken bieten einen umfassenden und intuitiven Einblick in die Transkriptionsprofile verschiedener gesunder und erkrankter Augengewebe. So verbessern sie unser Verständnis der zugrunde liegenden molekularen Krankheitsprozesse, unterstützen die Hypothesengenerierung und helfen bei der Suche nach neuen diagnostischen und therapeutischen Zielen für verschiedene Augenerkrankungen.

Next generation sequencing (NGS) enables the simultaneous sequencing of millions of DNA or RNA molecules and has revolutionized basic science and translational research in recent years, uncovering disease-relevant processes. While the genome describes the information of the DNA, which is identical in each cell, the transcriptome represents the total of all RNA molecules and is thus dynamic and varies between different cells and tissues. Transcriptome analysis using RNA sequencing thus allows determination of the functional state of a tissue and is increasingly applied in clinical routine, e.g., for diagnostic classification of cancers [9], estimation of cancer prognosis [28], and prediction of treatment response [7]. Large databases such as the *Cancer Genome Atlas *[6] provide the sequencing raw data generated in previous studies, although hardly any ocular tissue has been included so far. Moreover, the analysis of the raw data requires advanced bioinformatics skills. Therefore, in recent years, special web-based and user-friendly databases have been established, which allow intuitive exploration and comparative analysis of transcriptional profiles of ocular tissues. The aim of this review is to provide an overview of the currently available ocular transcriptome databases and to highlight their advantages and limitations.

## Principle of RNA sequencing

**Fig. 1:**
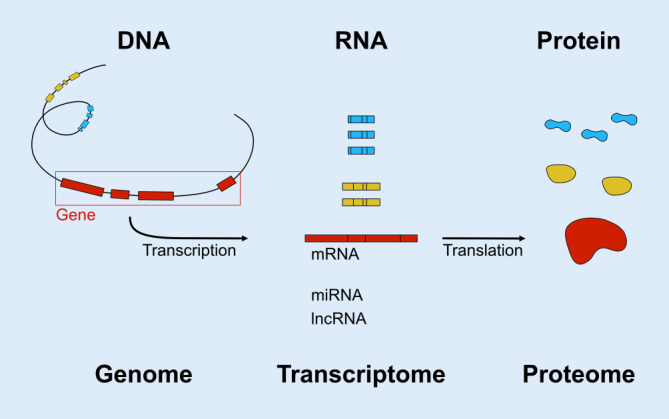
Path from genes to proteins. DNA is transcribed into RNA. RNA can serve as a template to produce proteins (translation, mRNA) or can exert regulatory functions in this process (miRNA, lncRNA). The complete sets of genes, RNAs, and proteins are referred to as the genome, the transcriptome, and the proteome, respectively. RNA sequencing allows the analysis of all RNA molecules contained in a sample

RNA sequencing allows the nucleotide sequences of millions of RNA molecules in a sample to be analyzed [24]. By comparing these sequences with the known reference genome, it is possible to identify and quantify different RNA molecules. The RNA serves as a template to produce proteins or can exert regulatory functions in this process. Thus, transcriptome analysis provides unbiased insights into the functional state of a tissue (Fig. 1).

Comparing samples from diseased and healthy tissues can provide detailed insights into the pathophysiology of a disease and can identify novel diagnostic and prognostic biomarkers. Thanks to the *Human Genome Project *[13] and technological advances, the costs and time of sequencing have significantly decreased in recent years, and it is likely that this trend will lead to an increased use of the technology in clinical routine [8]. In addition to unfixed samples, specialized sequencing methods can analyze the transcriptional profile of archived formalin-fixed and paraffin-embedded samples, greatly facilitating the analysis of rare diseases [2].

## Application in oncology

Transcriptome analysis has so far been used in particular in oncology [7, 9, 28]. For example, using transcriptome data from lung tumors and control tissue, diagnostic biomarkers were identified that enabled differentiation between tumor and control tissue with an accuracy of 98% in an independent validation dataset consisting of over 1000 tumors [9]. In addition, squamous cell and adenocarcinoma of the lung were differentiated with a classification accuracy of 95% [9]. Another example of an application of RNA sequencing in clinical routine is the estimation of tumor prognosis based on its transcriptional profile. Uhlen et al. analyzed the transcriptome of over 8000 samples of the most common cancer types and identified prognostically relevant biomarkers for each entity, which allowed the prognosis to be estimated [28]. The prediction of treatment response of a tumor also represents an interesting and clinically useful application of transcriptome analysis. The molecular characterization of various cancer types using RNA and DNA sequencing enabled classification across cancer types into four molecular subtypes with subtype-specific response rates to immune checkpoint inhibitor therapy, thus, providing a foundation for personalized cancer therapy [7]. A recently published statement of the German Medical Association (*Bundesärztekammer*) assumes that in the next few years, molecular tumor classification will become the standard procedure for most patients starting at the initial diagnosis with the aim to provide a precise and personalized treatment strategy [20].

## Application in ophthalmology

In ophthalmology, RNA sequencing has been comparatively rarely used so far, especially in clinical practice. Recently, a gene-expression-based diagnostic classification of conjunctival squamous cell carcinoma and papilloma was described [3, 15]. In addition, gene expression of specific cell receptors mediating SARS-CoV‑2 infection has been investigated in ocular surface tissues [14] and intraocular tissues [16] using RNA sequencing. Hyalocytes from the vitreous of patients with epiretinal membranes or macular holes were also recently characterized as an active and immunomodulatory cell population using RNA sequencing [4]. A prognostic gene expression signature for ocular tumors was successfully obtained for choroidal and conjunctival melanoma [21, 32]. Based on the transcriptional profile, uveal melanoma was classified into four prognostically relevant molecular subtypes [21]. This classification achieved a higher predictive power for distant metastases 5 years after brachytherapy than the traditional classification according to the *American Joint Committee on Cancer Staging Manual (8th Edition) *[17]. Likewise for conjunctival melanoma, 20 prognostically relevant biomarkers have been identified to estimate the risk of local recurrence or distant metastases [32]. For neovascular age-related macular degeneration (nAMD), RNA sequencing of choroidal neovascularization (CNV) membranes identified calprotectin (*S100A8/S100A9*) and secreted phosphoprotein 1 (*SPP1*) as novel nAMD-associated factors [22, 23, 31]. Intravitreal injection of an SPP1 inhibitor significantly modulated CNV size in the murine laser CNV model, highlighting the role of the factor as a potential new therapeutic target for nAMD [23].

## Transcriptome databases

With technological progress leading to a significant increase in transcriptome analyses, large databases containing a variety of publicly available transcriptome datasets of different diseases have emerged in recent years [6, 10]. One of the largest databases is the *Cancer Genome Atlas*, which to date contains the sequencing data of over 84,000 tumor samples from 67 different entities [6]. The diversity of these data has made it possible to catalog typical genetic and molecular alterations occurring in different tumors, both to increase knowledge of each tumor entity and to improve understanding of cross-entity mechanisms of carcinogenesis [11]. In addition, the raw sequencing data are publicly available and can be used, for example, as a validation dataset [9]. Reference should also be made at this point to the *Human Protein Atlas *[27], which catalogs human proteins in cells, tissues, and organs using a combination of various “omics” technologies, such as mass spectrometry and antibody-based proteomics. Despite the numerous possibilities mentioned above, the *Cancer Genome Atlas* does not yet include ocular tissues, with the exception of uveal melanoma. Although efficient algorithms exist to analyze the available raw sequencing data, they require advanced bioinformatics skills and are also relatively time-consuming. For these reasons, there is a need for databases that contain transcriptional profiles of ocular tissues while allowing intuitive data analysis.

## Ocular transcriptome databases

Here, we provide an overview of the available ocular transcriptome databases (Table 1).

**Table 1:** Overview of searchable ocular transcriptome databases

Database	*iSyTE 2.0*	*Express*	*retina.tigem.it*	*Spectacle*	*UCSC Cell* *Browser*	*Broad Institute* *Single Cell* *Portal*	*Eye in a Disk*	*Ocular Tissue Database*	*Human Eye Transcriptome Atlas*
Healthy tissue	Lens	Lens Retina	Retina	Retina RPE/Choroid	Retina RPE/Choroid Cornea	Cornea Iris TMW Sclera Retina RPE/Choroid	Cornea Lens Retina RPE/Choroid	Cornea Sclera TMW Iris Ciliary body Lens Optic nerve head Optic nerve Retina RPE/Choroid	Conjunctiva Cornea Lid Lacrimal gland Optic nerve Retina periphery Retina center RPE/Choroid ILM retinal Microglia Hyalocytes
Diseased tissue	–	–	–	Autoimmune retinopathy RPE nAMD	RPE nAMD	–	Retina AMD	–	Conj. SCC Conj. papilloma Conj. melanoma Pterygium CNV-membrane Epiretinal membrane PDR membrane PVR epiretinal PVR subretinal
Tissue types	1	2	1	4	4	6	5	10	20
Samples	42	56	50	23	23	18	829	6	139
Species	Murine	Murine	Human	Human	Human	Human, porcine	Human	Human	Human
Tissue source	Mouse	Mouse	Postmortal	Postmortal	Postmortal	Postmortal	Postmortal & stem cells	Postmortal	Surgical specimens
Method	Microarray	RNA-Seq	RNA-Seq	scRNA-Seq	scRNA-Seq	scRNA-Seq	RNA-Seq	Microarray	RNA-Seq
Methodological homogeneity^a^	No	No	Yes	No	No	No	No	Yes	Yes
Comparative analysis^b^	Yes	Yes	Yes	No	No	No	Yes	Yes	Yes
Link	https://research.bioinformatics.udel.edu/iSyTE	https://sysbio.sitehost.iu.edu/express	http://retina.tigem.it	http://singlecell-eye.com	https://cells.ucsc.edu/?bp=eye	https://singlecell.broadinstitute.org	https://eyeIntegration.nei.nih.gov	https://genome.uiowa.edu/otdb	https://www.eye-transcriptome.com
Reference	[11]	[5]	[18]	[29]	[24]	–	[25]	[30]	[33]

*AMD* age-related macular degeneration, *Conj.* conjunctiva, *CNV* choroidal neovascularization, *FFPE* formalin-fixed and paraffin-embedded, *ILM* internal limiting membrane, *nAMD* neovascular AMD, *PDR* proliferative diabetic retinopathy, *PVR* proliferative vitreoretinopathy, *RNA-Seq* RNA-sequencing, *RPE* retinal pigment epithelium, *scRNA-Seq* single-cell RNA-Seq, *TMW* trabecular meshwork, *UCSC* University of California, Santa Cruz

^a^Methodological homogeneity combines the following quality criteria: Confirmation of histological diagnosis by experienced ophthalmic pathologists, applying the same sequencing protocol for all samples to reduce technical variability

^b^Comparative analysis: all samples were integrated in the same bioinformatic model, allowing to normalize gene expression between different tissue types

### iSyTE and Express

The *iSyTE *(https://research.bioinformatics.udel.edu/iSyTE) [12] and *Express* (https://sysbio.sitehost.iu.edu/express) [5] databases provide the transcriptional profiles of murine lens and retina samples, including a wide range of embryonic and postnatal stages. This enables intuitive analysis and visualization of gene expression at different stages of lens and retina development. The raw data are largely derived from publicly available datasets generated by varying sequencing protocols at different institutions, therefore limiting these databases due to methodological inhomogeneity. In addition, microarray technology, which the *iSyTE database* is based on, is limited by higher technical variability compared to RNA sequencing, as well as by the lack of detection of rare and novel transcripts [18]. Moreover, microarray analyses can only detect those transcripts for which a corresponding probe is available, meaning that unlike RNA sequencing, it is not a completely unbiased analysis [18].

### retina.tigem.it

The *retina.tigem.it *database (http://retina.tigem.it) contains the transcriptional profiles of 50 healthy human retinas [19], thus, providing a comprehensive and intuitively searchable reference transcriptome dataset of the human retina. However, the samples are postmortem tissue, which is subject to rapid RNA degradation due to the prolonged period between death and preservation, thereby limiting the validity of the data [1, 22].

### Spectacle, UCSC Cell Browser, and Single Cell Portal

The *Spectacle *(http://singlecell-eye.com), *UCSC Cell Browser *(https://cells.ucsc.edu/?bp=eye), and *Single Cell Portal *(https://singlecell.broadinstitute.org) platforms enable exploration of single-cell RNA sequencing data from human retina, choroid/RPE, cornea, iris, trabecular meshwork, and scleral tissue, and also contain diseased tissue from patients with autoimmune retinopathy or neovascular AMD [29]. Even without bioinformatics expertise, the user can analyze which cell types express a specific gene and which subpopulations exist within a cell type, as well as explore cell type-specific marker genes. All three databases are based on postmortem tissue, thus, previously mentioned limitations need to be considered.

### Eye in a Disk

The *Eye in a Disk *database (https://eyeIntegration.nei.nih.gov) is currently the largest ocular transcriptome database with 829 samples in total [26], although relatively few different tissue types (retina, choroid/RPE, cornea, and lens) are available. It is the only database which allows comparison of ocular transcriptional profiles with non-ocular tissues. *Eye in a Disk *is limited by postmortem or stem cell-derived tissue and methodological inhomogeneity.

### Ocular Tissue Database

The *Ocular Tissue Database *(https://genome.uiowa.edu/otdb) provides the transcriptional profiles of a relatively large number of various healthy human ocular tissue types (10 entities) [30]. However, the database does not include diseased ocular entities and is also limited by microarray technology and postmortem tissue.

### Human Eye Transcriptome Atlas

**Fig. 2:**
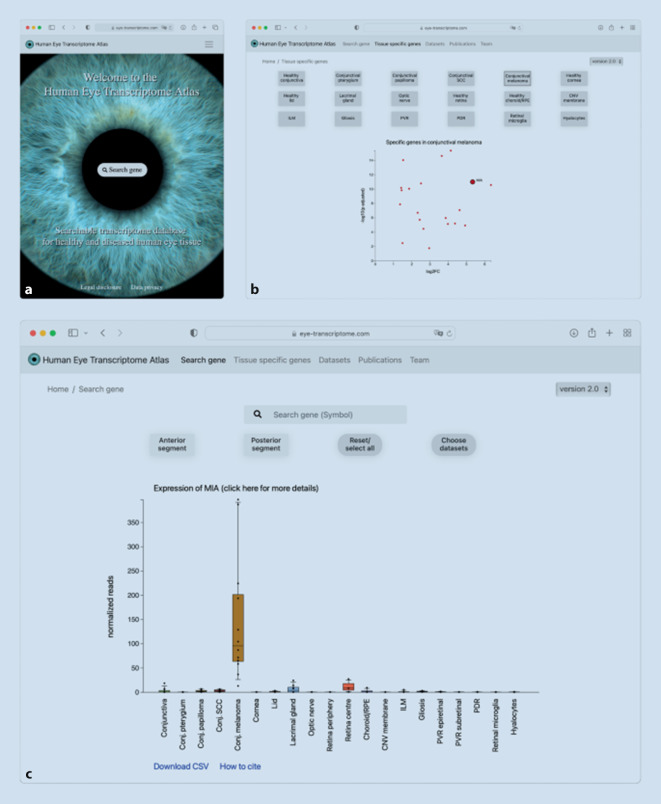
Exploring gene expression in eye tissues using the *Human Eye Transcriptome Atlas* (**a**). In addition to analyzing tissue-specific factors, as shown for *MIA *(melanoma inhibitory activity) for conjunctival melanoma (**b**), the user can visualize the expression of each gene in 20 different healthy and diseased ocular tissues without bioinformatics expertise (**c**). Tissues can be displayed by selecting categories (e.g., all tissues of the anterior or posterior segment of the eye or all healthy or diseased tissues) or can also be selected manually. By clicking the “Download CSV” button below the plot, the user can download the displayed expression values. In addition, the raw sequencing data are available under the *Datasets *tab. To obtain more information about the gene of interest, the user can click on the title of the plot, which links to the corresponding page of the gene in the *GeneCards *database*. *The *Human Eye Transcriptome Atlas *can be accessed via the following link: https://www.eye-transcriptome.com

The *Human Eye Transcriptome Atlas *which was recently developed by our group (https://www.eye-transcriptome.com, [33]) provides the largest number of different ocular tissue types of all currently available databases and contains the highest number of diseased ocular entities including conjunctival melanoma, conjunctival squamous cell carcinoma, conjunctival papilloma, pterygia, as well as epiretinal membranes, choroidal neovascular membranes from patients with neovascular AMD, retinal neovascular membranes from patients with proliferative diabetic retinopathy, and membranes from patients with proliferative vitreoretinopathy (epi- and subretinal) (Fig. 2). With a total of 139 transcriptome datasets, the *Human Eye Transcriptome Atlas is *one of the two largest databases and is the only database that, in contrast to databases describing postmortem tissue, contains surgically removed tissue samples that were either transferred to RNA stabilization solution or underwent FFPE (formalin-fixed and paraffin-embedded) processing immediately after surgical removal [2, 4]. This approach offers the advantage of reducing the rapid RNA degradation which occurs in postmortem samples [1, 22]. All samples included in the *Human Eye Transcriptome Atlas *were collected, processed and assessed by experienced ophthalmic pathologists at the same institution, and sequenced using the same sequencing protocol. This ensures a high standard of sample quality and also reduces technical variability between samples.

## Conclusion

Transcriptome databases such as the *Cancer Genome Atlas *[6] so far contain only very few ocular tissues and provide only the sequencing raw data, which require advanced bioinformatics skills to analyze. Therefore, specialized databases with different application focuses have emerged to provide transcriptional profiles of ocular tissues while enabling intuitive data analysis. Regarding the databases summarized in this review, *Spectacle, *the *UCSC Cell Browser, *and the Broad Institute’s *Single Cell Portal *allow intuitive exploration of single-cell RNA sequencing data from retina, choroid, cornea, iris, trabecular meshwork, and scleral tissues. The *Human Eye Transcriptome Atlas *provides the largest number of different ocular tissue types, contains the highest number of diseased ocular entities, and achieves a high standard of quality through methodological homogeneity. Ocular transcriptome databases provide comprehensive and intuitive insights into the transcriptional profiles of various ocular tissues and diseases, allowing rapid hypothesis testing in the search for new diagnostic and therapeutic targets.

## Declarations

For this article no studies with human participants or animals were performed by any of the authors. All studies mentioned were in accordance with the ethical standards indicated in each case.

The supplement containing this article is not sponsored by industry.
